# Supplementary material for: Inhibiting the mitochondrial pyruvate carrier does not ameliorate synucleinopathy in the absence of inflammation or metabolic deficits
Source: Free Neuropathol. Author manuscript; Available in PMC 2022 Feb 24. (PMC8870797; doi:10.17879/freeneuropathology-2020-3049)
Supplement: Supplementary Figures. Peelaerts et al. [file NIHMS1774579-supplement-Supplementary_Figures__Peelaerts_et_al_.pdf]

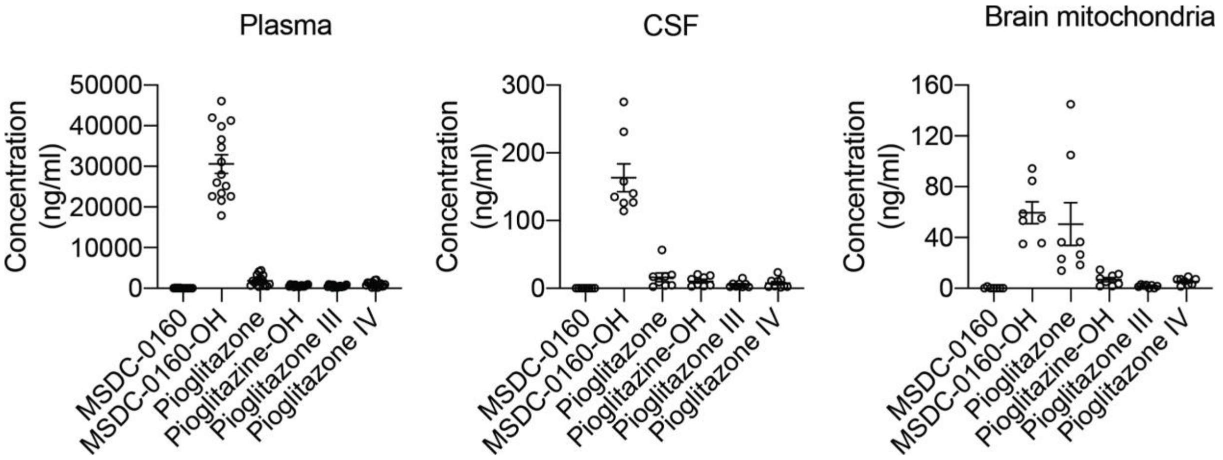

Supplementary figure 1. Levels of exposure of MSDC-0160 and pioglitazone and their metabolites in plasma, CSF and brain mitochondria. Concentration of MSDC-0160, pioglitazone and their metabolites were measured 18-20 hours after dosing by LC/MS. Dosing with equal amounts of MSDC-0160 or pioglitazone results in greater MSDC-0160-OH metabolite level in the CSF and rat brain mitochondria compared to pioglitazone metabolites (n = 16 for plasma, n = 8 for CSF and brain mitochondria, SEM).

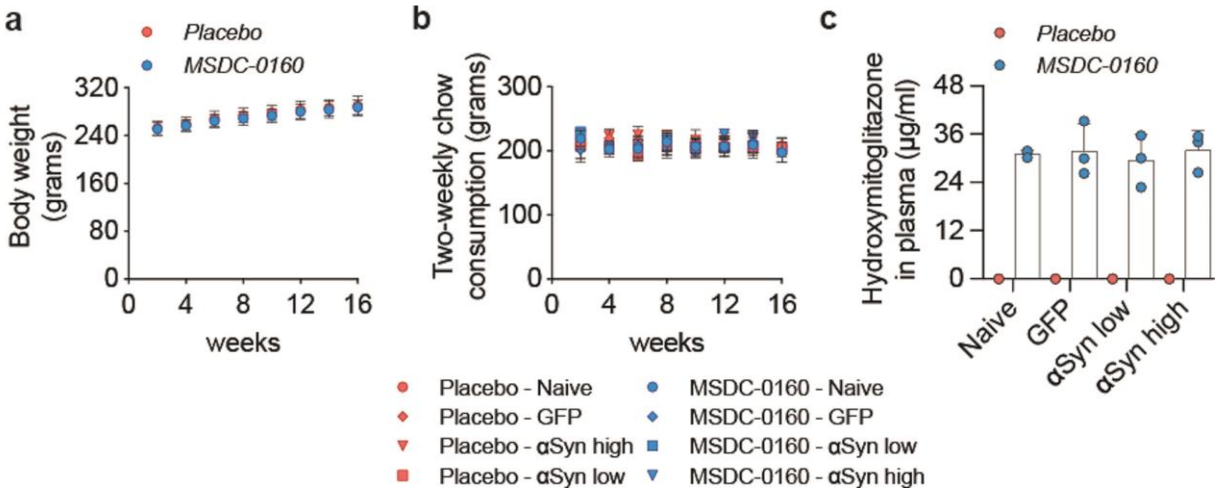

Supplementary Figure 2. Body weight, chow consumption and plasma MSDC-0160 metabolites. a) Body weight of female rats during 16 weeks of MSDC administration (n = 80, SEM) **b)** Chow consumption of placebo or MSDC-0160 infused chow. Intake was averaged over two weeks per animal (n = 20, SEM) **c)** Plasma levels of hydroxymitoglitazone, the active metabolite of MSDC-0160 in female rats, 16 weeks after start of MSDC-0160 administration. No metabolites were detected in placebo treated animals (n = 3, SEM).

Copyright: © 2020 The author(s). This is an open access article distributed under the terms of the Creative Commons Attribution 4.0 International License (https://creativecommons.org/licenses/by/4.0/), which permits unrestricted use, distribution, and reproduction in any medium, provided the original author and source are credited, a link to the Creative Commons license is provided, and any changes are indicated. The Creative Commons Public Domain Dedication waiver (https://creativecommons.org/publicdomain/zero/1.0/) applies to the data made available in this article, unless otherwise stated.

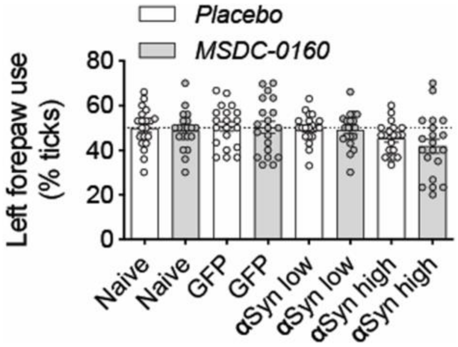

Supplementary Figure 3. Motor behavior analysis of MSDC-0160 treated animals. Cylinder test for αSyn overexpressing and control animals shows no significant changes or motor deficits in animals treated with MSDC-0160 (n = 40, SEM).

Copyright: © 2020 The author(s). This is an open access article distributed under the terms of the Creative Commons Attribution 4.0 International License (https://creativecommons.org/licenses/by/4.0/), which permits unrestricted use, distribution, and reproduction in any medium, provided the original author and source are credited, a link to the Creative Commons license is provided, and any changes are indicated. The Creative Commons Public Domain Dedication waiver (https://creativecommons.org/publicdomain/zero/1.0/) applies to the data made available in this article, unless otherwise stated.

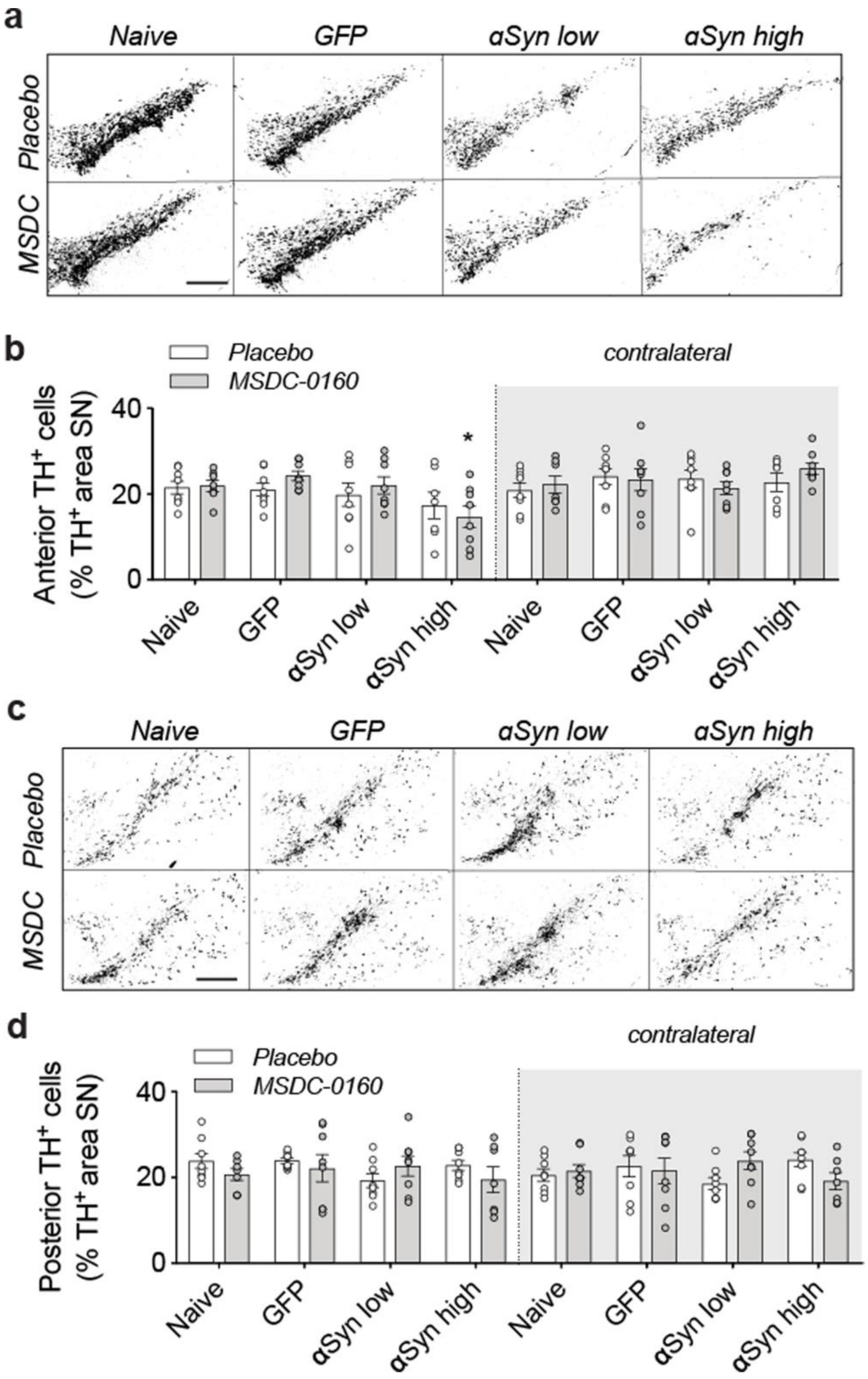

Copyright: © 2020 The author(s). This is an open access article distributed under the terms of the Creative Commons Attribution 4.0 International License (https://creativecommons.org/licenses/by/4.0/), which permits unrestricted use, distribution, and reproduction in any medium, provided the original author and source are credited, a link to the Creative Commons license is provided, and any changes are indicated. The Creative Commons Public Domain Dedication waiver (https://creativecommons.org/publicdomain/zero/1.0/) applies to the data made available in this article, unless otherwise stated.

Supplementary Figure 4. Nigral cell death anterior in SN. a) Fluorescent detection of TH-positive dopaminergic neurons in the ipsilateral anterior rat SN. Binarized images were set at a threshold by the adaptive triangle method in ImageJ. Scale bar indicates 500 µm. **b)** Quantification of TH positive area after thresholding shows significant cell loss in the anterior part of rat SN (n = 8, SEM, $p < 0.05$ for two-way ANOVA with Tukey post hoc correction compared to naïve condition treated with MSDC-0160). No differences between treatment conditions were detected. **c)** Fluorescent TH detection after adaptive triangle thresholding of ipsilateral posterior rat SN. Scale bar indicates 500 µm. **d)** Quantification of TH positive area after thresholding shows no cell loss in the posterior part of rat SN (n = 8, SEM).

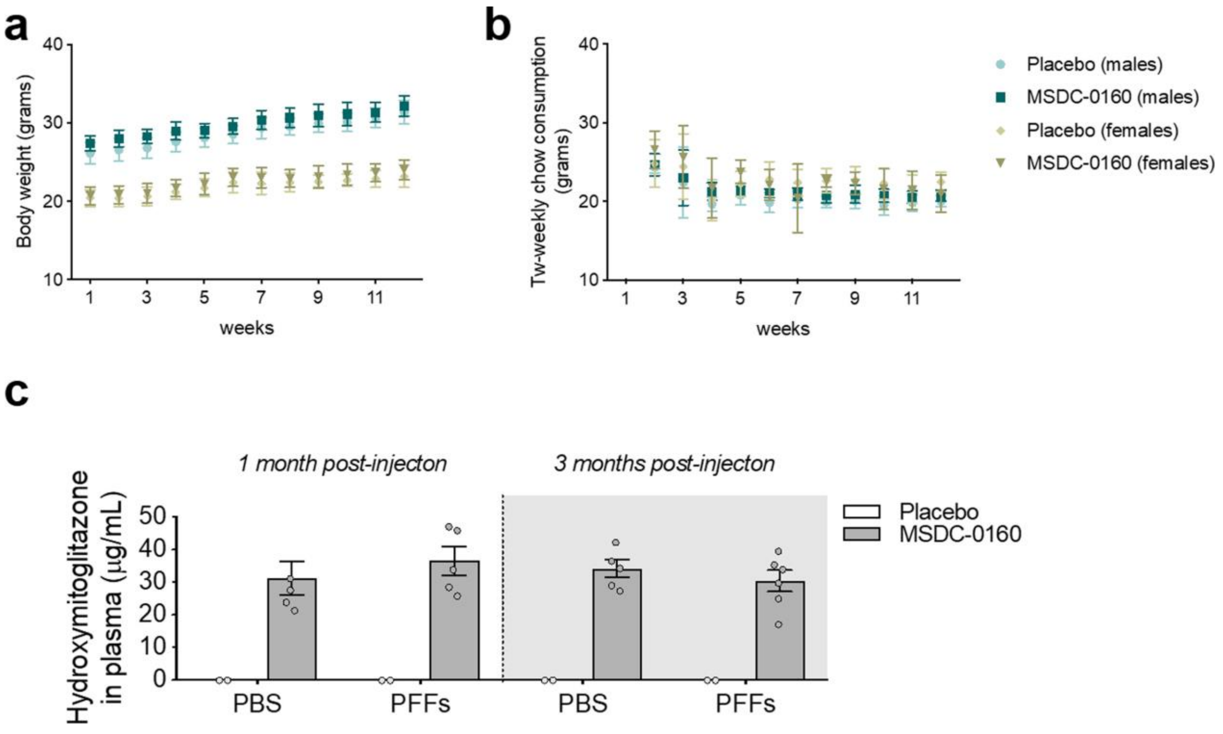

Supplementary Figure 5. Mouse body weight, chow consumption and plasma MSDC-0160 metabolites. **a)** Body weight of female and male mice during three months of MSDC-0160 or placebo administration (n = 9, SEM) **b)** Chow consumption of placebo or MSDC-0160 containing chow. Intake was averaged over two weeks per animal (n = 9, SEM) **c)** Plasma levels of hydroxymitoglitazone, the active metabolite of MSDC-0160 in female and male mice, 5 (n= 9, SEM) and 13 (n= 11, SEM) weeks p.i. No metabolites were detected in placebo treated animals (n = 8, SEM).

Copyright: © 2020 The author(s). This is an open access article distributed under the terms of the Creative Commons Attribution 4.0 International License (https://creativecommons.org/licenses/by/4.0/), which permits unrestricted use, distribution, and reproduction in any medium, provided the original author and source are credited, a link to the Creative Commons license is provided, and any changes are indicated. The Creative Commons Public Domain Dedication waiver (https://creativecommons.org/publicdomain/zero/1.0/) applies to the data made available in this article, unless otherwise stated.

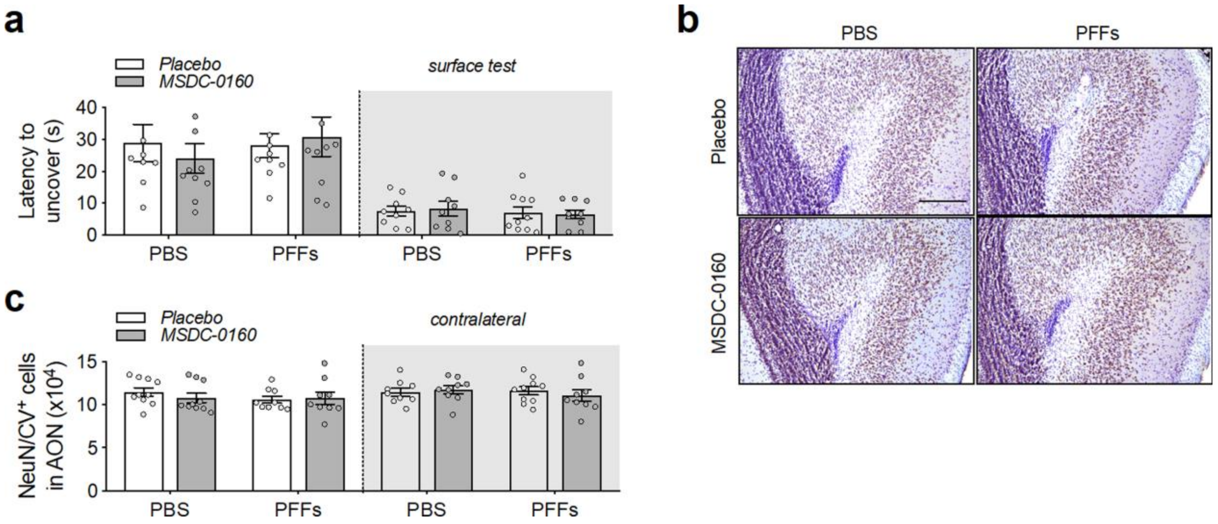

Supplementary Figure 6. Evaluation of olfactory function and neuronal cell survival in MSDC-0160 treated animals. **a)** Buried pellet testing in PFF and PBS animals showed no significant changes regardless of MSDC-0160 or placebo treatment (n = 9-10/group, SEM) 13 weeks p.i. **b)** Representative images of NeuN/CV staining in the ipsilateral AON. Scale bar = 250 µm. **c)** Stereological quantification of NeuN/CV positive cells in the AON 13 weeks p.i. (n = 9-10/group, SEM).

Copyright: © 2020 The author(s). This is an open access article distributed under the terms of the Creative Commons Attribution 4.0 International License (https://creativecommons.org/licenses/by/4.0/), which permits unrestricted use, distribution, and reproduction in any medium, provided the original author and source are credited, a link to the Creative Commons license is provided, and any changes are indicated. The Creative Commons Public Domain Dedication waiver (https://creativecommons.org/publicdomain/zero/1.0/) applies to the data made available in this article, unless otherwise stated.

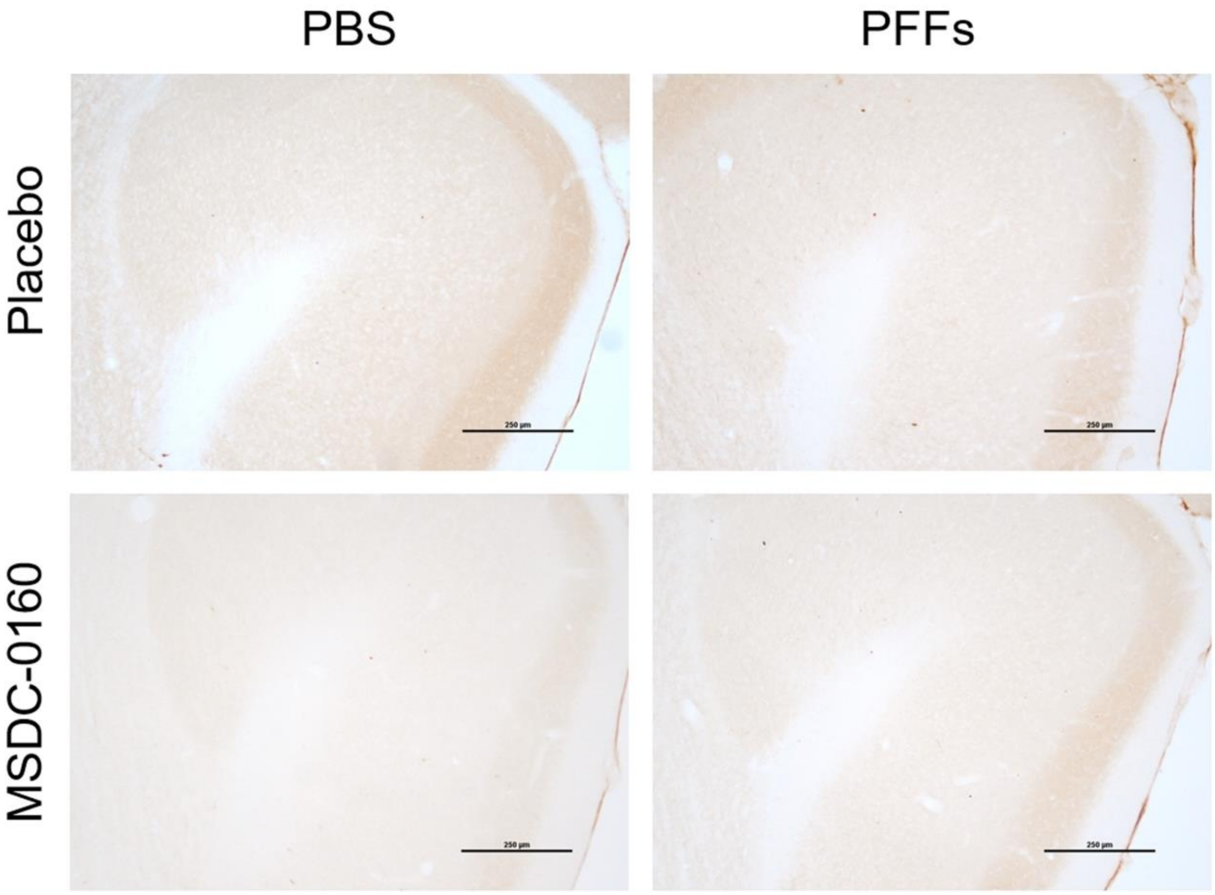

Supplementary Figure 7. No ni-αSyn detected in the PFF or PBS injected mice. Representative images showing no ni-αSyn positive staining in the AON 13 weeks p.i. (n = 3/group). Scale bar = 250 µm.

Copyright: © 2020 The author(s). This is an open access article distributed under the terms of the Creative Commons Attribution 4.0 International License (https://creativecommons.org/licenses/by/4.0/), which permits unrestricted use, distribution, and reproduction in any medium, provided the original author and source are credited, a link to the Creative Commons license is provided, and any changes are indicated. The Creative Commons Public Domain Dedication waiver (https://creativecommons.org/publicdomain/zero/1.0/) applies to the data made available in this article, unless otherwise stated.
